# Radiation Recall Dermatitis in Patients Treated with Sorafenib

**DOI:** 10.1155/2018/2171062

**Published:** 2018-01-24

**Authors:** Keyur Mehta, Andreas Kaubisch, Justin Tang, Aneesh Pirlamarla, Shalom Kalnicki

**Affiliations:** ^1^Department of Radiation Oncology, Montefiore Medical Center, Albert Einstein College of Medicine, Bronx, NY, USA; ^2^Department of Medical Oncology, Montefiore Medical Center, Albert Einstein College of Medicine, Bronx, NY, USA

## Abstract

**Introduction:**

Radiation recall dermatitis (RRD) is a phenomenon that occurs in previously irradiated areas shortly after administration of a chemotherapeutic agent. As the use of sorafenib expands, the incidence of radiation recall dermatitis induced by sorafenib will likely increase. Here, we report on a patient who developed RRD and describe his clinical characteristics along with a review of the literature.

**Case Presentation:**

Our patient was treated with palliative radiation therapy (RT) to a painful metastatic hepatocellular carcinoma lesion in the right forearm. He completed his radiation course with grade 1 dermatitis, which had resolved by the time he was started on sorafenib 400 mg twice daily 7 days afterwards. On the 21st day after RT, he presented with desquamation and erythema in the previously irradiated area of the right forearm, consistent with RRD. The sorafenib was discontinued and his symptoms subsequently resolved with conservative topical management.

**Conclusions:**

Although the pathophysiologic mechanism of sorafenib-related radiation recall dermatitis remains to be investigated, practitioners should be aware of its presence and management in order to improve clinical outcomes.

## 1. Introduction

Radiation recall dermatitis (RRD) is a phenomenon that occurs in previously irradiated areas shortly after administration of a chemotherapeutic agent. Radiation recall dermatitis has been reported with usage of cisplatin, docetaxel, and trastuzumab. Sorafenib is a tyrosine kinase inhibitor that has been commonly used in locally advanced and metastatic hepatocellular carcinoma (HCC) with phase III clinical trial showing improvement in overall survival in patients with inoperable HCC [1].

Application of radiation therapy in HCC has increased with the advent of advanced radiation planning techniques such as stereotactic body radiation therapy. As a result, the clinical application of radiation therapy with sorafenib continues to increase. While the side-effect profile of sorafenib as monotherapy has been well described, the cumulative effects with radiation therapy are less clear. RRD triggered by sorafenib is rare, and only few cases have been reported [2, 3]. Here, we report a case of sorafenib-induced radiation recall dermatitis along with a review of the literature.

## 2. Case Report

**Figure 1:**
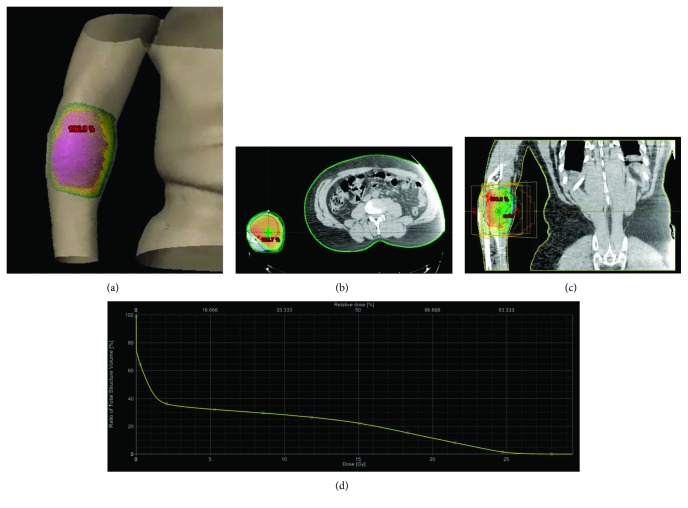
Radiotherapy planning with two oblique fields. Radiotherapy field with (a) three-dimensional model view, (b) axial view, and (c) coronal view. (d) Dose-volume curve demonstrates that the skin *D*_50%_ was 15.2 Gy. Mean dose of skin was 13.1 Gy, and the maximum dose was 29.4 Gy.

A 59-year-old man with hepatitis C presented with an enlarging right forearm lesion that was gradually becoming more painful. Biopsy of the painful lesion showed metastatic hepatocellular carcinoma. On PET/CT imaging, he was shown to have several hepatic lesions and a FDG avid lesion in the right forearm. His right forearm lesion received radiofrequency ablation and was followed by palliative radiation therapy. The patient received a total dose of 30 Gy in 10 fractions using two oblique fields (Figure 1). The mean dose of the skin was 13.1 Gy, and the maximum dose was 29.4 Gy. The skin contour extended 3 cm superior and inferior to the gross tumor volume. The skin *D*_50%_ was 15.2 Gy. The patient's tumor decreased in size with improvement in his range of motion. He developed grade 1 dermatitis while receiving radiation therapy, but this had resolved prior to initiation of sorafenib.

**Figure 2:**
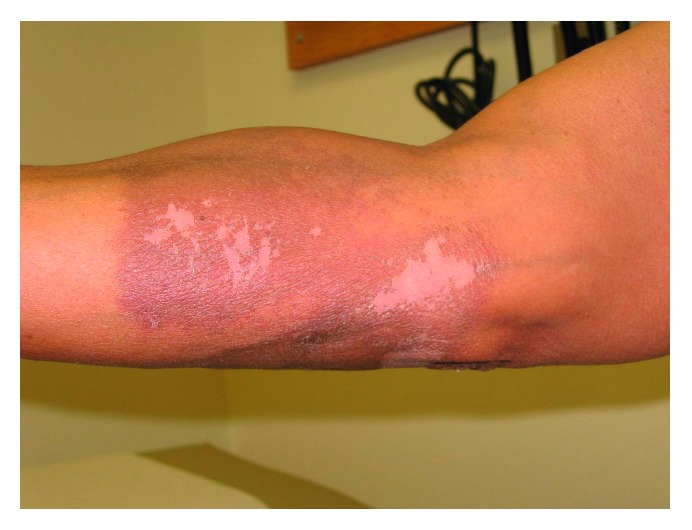
RRD demonstrated on previously irradiated area of the right forearm. RRD, radiation recall dermatitis.

The patient was started on sorafenib 400 mg twice daily after resolution of his grade 1 dermatitis and seven days after radiation therapy. When evaluated in follow-up 21 days after completion of radiation therapy, he was noted to have marked erythema and dry desquamation in the previously irradiated area with sharp demarcation of the adjacent skin, consistent with RRD (Figure 2). He did not have pruritus, pain, or tenderness associated with the RRD. He was determined to have grade 1 RRD.

The sorafenib was held, and the patient was started on topical antibiotics for prophylaxis and topical steroids for symptom management. Shortly afterwards, his dermatitis improved, and the patient was restarted on reduced dose sorafenib 200 mg daily with no further complications.

## 3. Discussion

The multimodality approach to cancer patients often involves a combination of chemotherapy and radiation therapy in a concomitant or sequential course. Radiation recall reactions are inflammatory states triggered by a systemic agent in a previously irradiated field. These reactions can manifest in internal organs and muscles but are most commonly seen as dermatologic effects. RRD has been described as a side effect for many systemic agents, such as docetaxel, paclitaxel, gemcitabine, pemetrexed, doxorubicin, and tamoxifen [4]. To date, RRD with sorafenib treatment has only been described in five other cases [2, 3, 5, 6].

In our patient, the skin reaction was observed 21 days after completion of radiation therapy. The acute reaction that was observed shortly after completion of radiation therapy was likely due to acute radiation dermatitis. However, that quickly resolved by itself. The second manifestation of dermatitis in the same area after initiation of sorafenib is more consistent with RRD. While the affected skin appears to have completely healed during the short one-week interval between RT and exposure of sorafenib, it is possible that there may be subdermal effects that are still in the process of healing. Therefore, it is possible that the presentation may also have a component of acute radiation dermatitis due to sensitization by sorafenib.

RRD typically appears after a dermatitis-free interval subsequent to the completion of radiation treatments. The possibility of RRD should be considered in anyone who developed a skin reaction in a previously irradiated area that was normal prior to the initiation of a new drug. This is inclusive of patients with distant history of radiation therapy, as the radiation-to-drug interval can range from days to years, who were administered a dose between 10 and 81 Gy [7].

The presentation of RRD is similar to the dermatologic reaction seen during active radiation therapy, namely, erythema, edema, desquamation, hemorrhage, and necrosis. These can be categorized in the RTOG acute toxicity definition of radiation dermatitis. However, Camidge and Price fashioned a new categorization of RRD that includes pruritus, urticaria, and vesiculation (Table 1) [8].

The exact mechanism of RRD is unknown, but hypotheses have focused on vascular damage, drug hypersensitivity, or stem cell inadequacy or sensitivity [7–9]. The histopathologic findings of RRD were described by Smith et al. in 10 patients who underwent a biopsy of affected areas [10]. Included in their findings were descriptions of hyperkeratosis, epidermal acanthosis, disordered proliferation with increased mitotic figures, follicular hyperkeratosis, follicular pustules, psoriasiform changes, dermal sclerosis, vascular dilatation, endothelial atypia, and stromal cell atypia [10].

The RRD described in our patient is typical of the recall reactions demonstrated with systemic agents that have been in use for many years. Our patient was managed with topical antibiotics for prophylaxis and topical steroids for inflammation. The general management of RRD is to discontinue the offending agents and offer supportive care as most symptoms self-resolve with time. Therefore, the prompt recognition of RRD and the offending agent is important in management.

## 4. Conclusion

Since sorafenib is used in patients with advanced carcinomas, it is not unlikely that these patients have received or will receive radiation therapy for primary or metastatic lesions. Although our patient had only mild dermatologic reactions, other agents have resulted in grade 3 and 4 RRD that require swift attention. This report of radiation recall dermatitis associated with sorafenib should prompt clinicians to carefully question and examine their patients who are at risk of RRD.

## Figures and Tables

**Table 1:** Radiation recall dermatitis toxicity grading.

Grade	Reaction
1	Erythema ± pruritus ± dry desquamation
2	Pain ± edema ± uricaria or vesiculation
3	Moist desquamation
4	Necrosis, ulceration or hemorrhage
